# Leachate from Weathered Face Masks Increases DNA Damage to Sperm of Sand Dollars *Scaphechinus mirabilis*

**DOI:** 10.3390/toxics13050372

**Published:** 2025-05-04

**Authors:** Victor Pavlovich Chelomin, Andrey Alexandrovich Mazur, Valentina Vladimirovna Slobodskova, Nadezhda Vladimirovna Dovzhenko, Sergey Petrovich Kukla

**Affiliations:** V.I. Il’ichev Pacific Oceanological Institute, Far Eastern Branch, Russian Academy of Sciences, Vladivostok 690041, Russia; chelomin@poi.dvo.ru (V.P.C.); slobodskova@list.ru (V.V.S.); doreme_07@mail.ru (N.V.D.); kukla.sp@mail.ru (S.P.K.)

**Keywords:** face mask, genotoxicity, leachate, microplastics, *Scaphechinus mirabilis*

## Abstract

The COVID-19 pandemic has exacerbated the problem of environmental contamination of disposable personal protective equipment, in particular face masks (FMs). As a result of environmental factors, FMs undergo aging and fragmentation processes and become a source of microplastics (MPs) and chemical additives. Taking into account the scale of accumulation of used FMs and their fragments in the coastal zone, it should be expected that the most appreciable ecotoxicological consequences should be observed in hydrobionts inhabiting coastal ecosystems. Based on this, the aim of this study was to investigate the toxicity of leachates from pristine and weathered FMs using sperm of sand dollars *Scaphechinus mirabilis*. In our work, we used pristine and weathered FMs, which for 200 days were kept in the external environment under natural conditions and exposed to a complex of climatic factors. Fourier transform infrared spectroscopy was used to characterize the chemical changes that occurred in the polymer structure of FMs during this period. It follows from the results obtained that leachates from pristine and weathered FMs reduced sperm viability; stimulated the accumulation of lipid peroxidation products, such as malondialdehyde (MDA); and significantly increased the destruction of DNA molecules, showing a genotoxic effect. Overall, our results complement the limited experimental data presented, indicating the genotoxic properties of face mask extracts.

## 1. Introduction

Studies in recent years have convincingly shown that synthetic polymers (plastics) are increasingly spreading in the biosphere and have entered not only hard-to-reach ecosystems but also food chains [1]. As the spread of plastic waste in the biosphere has been studied, it has become evident that the marine environment, and particularly coastal ecosystems, are most under pressure from various synthetic polymers. This trend is particularly evident in the case of plastic waste forming huge accumulations along the shoreline in the intertidal zone, which is essentially a transit zone for the migration of all plastic waste from terrestrial to oceanic environments [2,3,4,5]. Concentration of plastic debris in the intertidal zone is shaped by a variety of factors including population density, presence of waterways, complex climatic and hydrological conditions, coastal currents, and sea level fluctuations. Therefore, the accumulation of huge masses of plastic waste in the intertidal zone, which is a powerful source of nano- and microplastics and a complex set of chemicals, poses a threat to marine organisms and, above all, to littoral inhabitants [4,6,7,8,9,10].

The COVID-19 pandemic that swept the world, along with social and medical problems, further exacerbated this environmental problem [11,12,13,14,15]. To control the spread of viruses, a huge amount of personal protective equipment was produced in a relatively short period of time, dominated by disposable face masks (FMs) composed of aliphatic polymers, mainly polypropylene (PP). At the height of the pandemic, monthly production of FMs reached 129 billion pieces [16,17], and an estimate of the total annual amount of FMs used in 36 countries was more than 1.5 million tons [18].

Due to poorly developed disposal systems, in a short period, this personal protective equipment have become one of the most common types of litter and spread in terrestrial and aquatic environments [5,19]. According to conservative estimates, more than 1.56 billion FMs ended up in the oceans during the initial period of the pandemic alone [20]. Although masks are still found in all public places and even in remote ecosystems (e.g., uninhabited islands—Soko, Hong Kong), the coastal zone also bore the brunt of the FMs abundance. The density of FMs distribution in this zone, including the coastline and especially public places and beaches, river estuaries, closed and semi-closed lagoons, and bays, significantly exceeds any other place [5,11,14,17]. As a result of the natural factors characteristic of this zone, masks undergo intense “aging” processes, fragment and become, in addition to the actual pollution, a source of MPs and chemical additives [4,8,17,18,21]. It has been found that mechanical exposure releases several thousand microfibers and 10^8^ nanoplastics from a single mask into the environment [4], and after UV irradiation (180 h), the mask becomes a source of up to 173,000 microfibers/day [22]. In general, it is believed that a set of influences (agitation, mechanical forces, UV) results in the formation of several tens of thousands to hundreds of millions of differently sized plastics from a single mask [13]. Moreover, it has been shown that FMs fragments entering into the aquatic environment can serve as a substrate for the formation of biofilms of non-typical for this environment [23] or pathogenic [24] bacteria.

In addition, studies involving used FMs show that leachates from them have a profound effect on various cellular processes in a wide range of organisms, including phyto- and zooplankton [6,25,26], selected invertebrates [27,28], fish [29,30], and even human cells [31]. Taking into account the scale of accumulation of used FMs and their fragments in the coastal zone and the examples given above, it should be expected that the most noticeable ecotoxicological effects should be observed in hydrobionts living in littoral ecosystems, especially in river estuaries and ecosystems with reduced water exchange (lagoons and bays). Meanwhile, littoral ecosystems are not only a zone of the highest biological productivity, but also a place where numerous species of hydrobionts, including those of commercial importance, carry out the most important stages of their life cycle (i.e., spawning, fertilization, larval development).

Typical representatives of these hydrobionts are sea urchins, in particular the Far Eastern species of sand dollars *Scaphechinus mirabilis*. Specimens of this species, as a rule, spawn in shallow, well-warmed coastal waters, and their gametes are directly exposed to a variety of pollutants from terrigenous runoff, including plastic debris. This allows us to consider sea urchin gametes as a unique model for studying the impact of anthropogenic pressures, including the FMs used, on nuclear DNA stability, which is an important aspect of successful reproduction of any species. To develop this direction, as a first step, we set out to identify the vulnerability of flat sand dollar spermatozoa using traditional cytotoxic markers (resazurin assay and lipid peroxidation measurement) and a genotoxic assay (comet assay), thereby determining and comparing the degree of accessibility of *S. mirabilis* sperm DNA with labile components present in pristine (unused) and weathered FMs that are easily leached into the environment.

## 2. Materials and Methods

### 2.1. Preparation of Experimental Solutions from Masks

Three-layer face masks manufactured by Kazan factory SpetsMedZashchita (St. Petersburg, Russia) were used in this work. We experimentally reproduced the weathering of the masks outdoors. During the period from February to July (200 days in total), the FMs were in the external environment (42°58′42.8″ N, 131°43′37.6″ E). The climate in this area is maritime monsoonal, characterized by seasonal atmospheric circulation. Due to the boundary position between the Eurasian landmass and the Pacific Ocean, the region experiences active cyclonic and anticyclonic activity, the formation of frontal zones and, as a consequence, rapid changes in the weather regime, with a sharp change in the intensity of climatic factors. February–March—negative temperatures at night and daytime (from −20 °C to −6 °C); March–April—negative temperatures at night (from −6 °C to 0 °C) and positive temperatures during the day (from +3 °C to +9 °C); April–May—nighttime temperatures from +2 °C to +7 °C, daytime temperatures from +9 °C to +15 °C; May–June—nighttime temperatures from +7 °C to +12 °C, daytime temperatures from 15 °C to 18 °C (https://www.ncei.noaa.gov/access/search/data-search/global-hourly, accessed on 20 February 2025). The masks were located in an open area, fixed on a tripod, so insolation of the masks was full-fledged. In addition to temperature fluctuations and UV irradiation, the masks were affected by such factors such as air humidity, precipitation, and wind.

The pristine FMs were kept indoors without access to sunlight at a constant temperature of 20 °C ± 1 °C.

In laboratory conditions, the masks were divided into 2 groups—control (pristine masks) and aged (weathered) masks. Extractions from the masks were carried out in glass tanks with a volume of 5 l. In each of the tanks, 20 FMs were placed in 3 L of sterile seawater. For more successful leaching of additives, we cut the outer layer of the mask measuring 9.5 cm wide and 17.5 cm long into smaller fragments with 2 cm sides. For efficient extraction of chemicals from the masks, we placed the tanks on S-3.02L.A10 orbital shakers (Elmi, Riga, Latvia). The rotation speed of the platform was 100 RPM, and the extraction time was 96 h at 20 °C ± 1 °C. The leachates were filtered using Whatman GF/F glass fiber filters (d = 90 mm, pore size = 0.7 µm) (Maidstone, UK) in glass funnels. After filtration, parameters such as salinity, oxygen saturation, and Ph (31 ‰, oxygen richness 99–100%, pH 8 (IP67 Combo, AZ instrument Corp., Taichung, Taiwan ROC ) were measured. Then filtered samples were filled into dark glass bottles and frozen until the start of the experiment [32].

The leachates were used for the experiment at dilutions of 25%, 50%, 75%, and 100%.

### 2.2. Fourier Transform Infrared (FTIR) Spectroscopy

The FTIR spectra of both types of FMs fragments were determined using the IRAffinity-1S instrument (Shimadzu, Kyoto, Japan) with LabSolutions IR 2.27 software (Shimadzu, Kyoto, Japan). The instrument settings utilized for the purpose of determining the spectra have been delineated in a preceding publication [7]. The calculation of the content of functional groups in the samples was conducted using the following indexes: carbonyl index (CI) [33], hydroxyl index (HI), and carboxyl index (COI) [34].

### 2.3. Description of the Experiments

For the experiment, adult sand dollars *S. mirabilis* were collected in the Alekseev Bay of Popov Island (Peter the Great Bay, Sea of Japan) from a depth of 4 to 4.5 m, then placed in thermo-containers (8–10 °C) without water and transported to the laboratory within 30 min. Alekseev Bay is located in the center of Peter the Great Bay and extends into the northwestern shore of Popov Island in the southwestern part of Amur Bay. The upper part of the bay is characterized by shallow depths; in the middle part, the depth reaches 11 m and increases uniformly to 20 m towards the outlet. Popov Island is located at a considerable distance (more than 10 km) from sources of industrial and economic human activity. In addition, part of the territory of the island and the adjacent water area is occupied by the Far Eastern Marine Reserve, which is engaged in the protection of biodiversity. After delivery to the laboratory at the Popov Island Marine Experimental Station of POI FEB RAS, the animals were acclimatized at a temperature of 18–19 °C for 2 d. Then, gametes of *S. mirabilis* were obtained by stimulation of spawning with 0.5 M KCl solution. The eggs were processed according to the standard technique [35]. Sperm concentrate was collected immediately before the experiment and diluted with sterile seawater. Sperm concentrate from each male was collected in 10 mL of sterile seawater; then, the concentrate was diluted at a ratio of 1 mL of sperm to 9 mL of seawater. Control fertilization was then performed to check the quality of the germ cells; eggs with a fertilization rate below 95% were not used. The ratio of spermatozoa to oocytes was 200:1 in all experiments.

To evaluate the toxicity of the mask leachates, spermatozoa were incubated for 1 h in the test solutions at temperature 18 °C according to protocol EPS 1/RM/27 [36]. Fertilization was then carried out in pure sterile water, and the proportion of zygotes formed was counted after 20 min. The effect of FMs leachates was visually assessed by the formation of the fertilization membrane. Counting was performed in 4 parental pairs each in 3 parallels (n = 12) containing at least 100 zygotes. A graphic representation of the spermiotoxicity test procedure is provided in the Supplementary Materials (Figure S1 in Supplementary Materials).

### 2.4. Determination of the DNA Damage

After exposure, the extent of damage to DNA molecules was assessed in *S. mirabilis* sperm using an alkaline version of the DNA comet assay described previously [37]. The main procedures of the method consisted of immobilizing the post-incubated spermatozoa in low-melting 1% agarose (MP Biomedicals, Eschwege, Germany) and applying it to a slide. The slides were pre-coated with 1% normal-melting agarose (MP Biomedicals, Eschwege, Germany). Next, cell membrane lysis and protein extraction were performed in high-salt solution (2.5 M NaCl; 0.1 M EDTA-Na_2_; 1% Triton X-100; 10% DMSO; 0.02 M Tris-HCl, pH 10) for 1 h. After lysis, slides were subjected to alkaline treatment (300 mM NaOH, 1 mM EDTA-Na_2_) to denature DNA for 40 min (to detect DNA single-strand breaks and alkali-labile sites), followed by electrophoresis in alkaline medium at 2 V/cm for 20 min. After electrophoresis, slides were neutralized (0.4 M Tris-HCl, pH 7.4), fixed with ethanol, and stained with SYBR Green I.

Visualization of prepared and stained slides was performed using fluorescence microscopy in the fluorescence mode of the AxioImager A1 microscope (Carl Zeiss, Oberkochen, Germany) with a high-sensitivity AxioCam MRc camera (Carl Zeiss, Oberkochen, Germany). The comets were processed using specialized software V 1.2.2. CASP (Wroclaw, Poland, https://casplab.com accessed on 20 March 2025), determining the percentage of DNA in the “comet tail”. Data were processed on stored digital images. Apoptotic cells visualized on micrographs as weakly fluorescent DNA comets with a broad diffuse “tail” and almost absent “head” were excluded from the analysis. Counts were performed in 4 parental pairs in 3 parallels; each parallel contained 50 comets (n = 600).

### 2.5. Determination of Malondialdehyde Content

The content of malondialdehyde (MDA), a product of lipid peroxidation, in cell fractions was determined by color reaction with 2-thiobarbituric acid (TBA) [38]. Sperm was diluted in 1 mL of filtered sterile seawater. To 0.75 mL of the suspension, 0.5 mL of 30% trichloroacetic acid (TCA) and 0.5 mL of 0.75% TBA were added sequentially. The mixture was mixed thoroughly and thermostated on a water bath at 95 °C for 20 min. After thermostating, the samples were rapidly cooled and centrifuged at 3000 rpm for 30 min on a centrifuge. The optical density of the samples was measured on a Shimadzu UV-2550 spectrophotometer (Kyoto, Japan) at wavelengths of 580 nm and 532 nm. The molar extinction coefficient = 1.56 × 10^5^ cm^−1^ M^−1^ was used in the calculation of MDA concentration and expressed in μmol/mL.

### 2.6. Resazurin Cytotoxicity Assay

Determination of the ability of *S. mirabilis* sperm, after exposure to different concentrations of FMs leachates, to convert the dye resazurin (blue non-fluorescent) to resorufin (pink fluorescent) was measured using the test previously described by Knapp et al. [39].

A total of 500 µL of incubated *S. mirabilis* sperm was sampled for each study. Then, 50 µL of a 10-fold solution of resazurin in PBS (pH 7.4) was added to sperm and incubated for 1 h at 19 °C on a TS-100C thermoshaker (Biosan, Riga, Latvia). For colorimetric analysis, absorbance at 570 nm and at 600 nm was measured using a UV-2550 spectrophotometer (Shimadzu, Kyoto, Japan).

### 2.7. Statistical Analysis

The experiment results were processed in the MS Excel and Statistica 10 software packages (StatSoft, Tulsa, OK, USA). For the data, nonparametric Kruskal–Wallis ANOVA followed by pairwise Mann–Whitney tests were performed. A difference at *p* < 0.05 was considered to be statistically significant.

## 3. Results

### 3.1. Characterization of FMs Using FTIR

**Figure 1:**
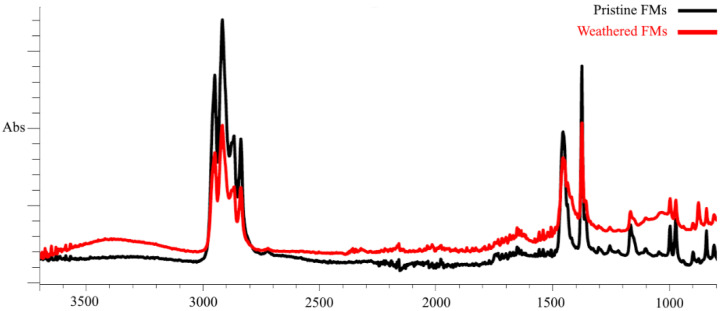
FTIR spectra of outer layer fragments of intact and weathered FMs.

In our work, we used pristine (unused) FMs and FMs that were in the external environment for 200 days (February–July) and that were exposed to a complex of climatic factors (mainly temperature and sunlight fluctuations). To characterize the chemical changes that occurred in the polymer structure of FMs during this period, the method of Fourier transform infrared spectroscopy was applied. Figure 1 shows the spectra of surface functional groups of pristine and weathered FMs.

As demonstrated by FTIR data, the outermost layer of FMs is constituted by polypropylene (PS) and exhibited two groups of spectral bands that are characteristic of this polymer. These bands were observed in the ranges of 3000–2800 cm^−1^ and 1500–1300 cm^−1^, corresponding to the stretching and bending vibrations of the C–H bond of alkanes, respectively. Following prolonged habitation of FMs in natural conditions and continuous exposure to climatic factors, substantial alterations are evident in the IR spectra of the outer layer, attributable to the emergence of novel peaks within the frequency ranges of 3600–3200 cm^−1^ and 1750–1650 cm^−1^. It is evident that these spectral peaks are indicative of the stretching of both the OH bond (hydroxyl group) and the valence C=O bond (carbonyl groups) oscillation. Furthermore, a quantitative analysis of the IR spectra of the outer layer of the experimental masks reveals significant alterations in the main spectral peaks within the frequency ranges of 3000–2800 cm^−1^ and 1500–1300 cm^−1^.

To quantify the changes observed in the polymer chains of experimental FMs, we quantitatively calculated the content of functional groups and presented them in the form of the corresponding indexes—CI (carbonyl), HI (hydroxyl), and COI (carboxyl). The calculated values of these indexes for the outer layer of pristine and experimental FMs are summarized in Table 1.

The obtained index values show that, compared with the unused FMs, significant changes in the chemical composition occurred in the surface chains of the experimental masks. This was most clearly expressed in the increase in carbonyl (almost six times) and hydroxyl (4 times) indexes, while the carboxyl index increased by almost only 30%.

### 3.2. Characterization of Sperm

#### 3.2.1. Sperm Viability

**Figure 2:**
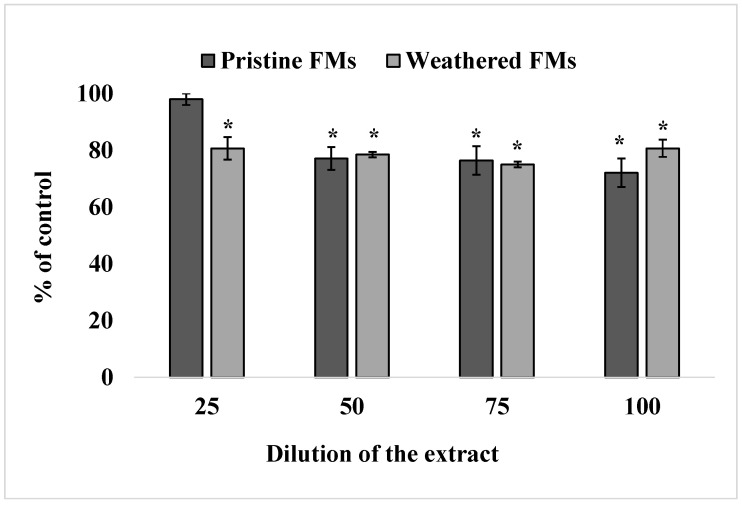
Effect of exposure to different concentrations of leachates from pristine and weathered face masks on the reduction of resazurin to resorufin (resazurin test) (mean ± standard deviation, n = 12); *—difference from the control is significant at *p* < 0.05.

The results of viability analysis of sand dollars sperm exposed to different concentrations of leachates from unused and experimental FMs are shown in Figure 2.

The resazurin test showed that leachates from both FMs significantly reduced the ability of *S. mirabilis* sperm to reduce resazurin into resorufin, indicating a decrease in their metabolic activity upon such treatment. In addition, it follows from the results of these experiments that leachates from weathered FMs caused an inhibitory effect at 25% dilution, while the corresponding extracts from unused FMs caused an effect only from 50% dilution on.

#### 3.2.2. MDA Content in Sperm

**Figure 3:**
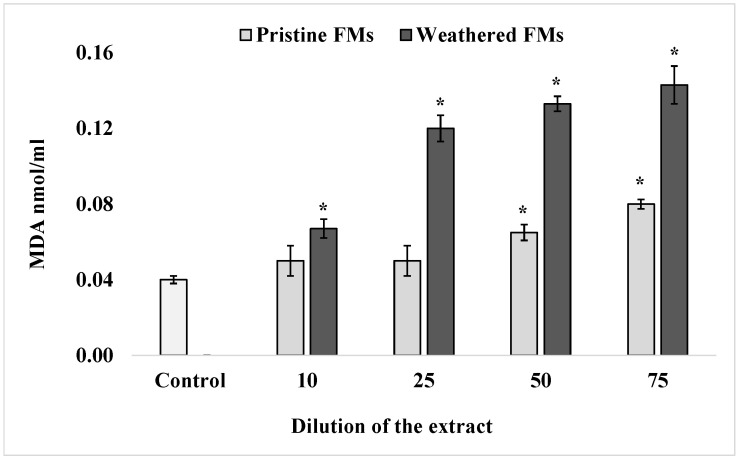
MDA levels in sperm *S. mirabilis* after exposure to different concentrations of leachates from pristine and weathered face masks (mean ± standard deviation, n = 12); *—difference from the control is significant at *p* < 0.05.

To determine the content of one of the end products of sperm lipid peroxidation, MDA, a widely accepted thiobarbituric acid method, was used. The results of these determinations, summarized in Figure 3, show that after exposure of *S. mirabilis* sperm to different concentrations of leachates from both FMs samples, the content of TBA-reactive products in lipids increased significantly in a dose-dependent manner. It is important to note that in the presented experiments, leachates from weathered FMs had a greater (more than 2-fold) effect on lipid peroxidation activation compared with extracts from pristine FMs samples.

#### 3.2.3. Genotoxicity

**Figure 4:**
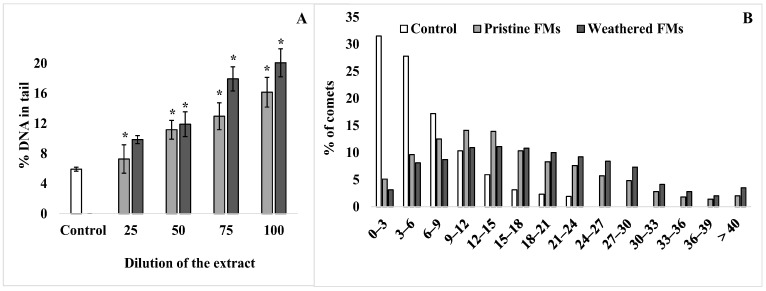
DNA damage of sperm *S. mirabilis* after exposure to different concentrations of leachates from pristine and weathered face masks. Value of % DNA in the comet tail (**A**) and distribution of comets according to the degree of fragmentation with an interval of 3% (**B**) (mean ± standard deviation, n = 12); *—difference from control is significant at *p* < 0.05.

To assess the level of destructive processes in the sperm genome of the sand dollars *S. mirabilis* after exposure to leachates from both FMs, the DNA comet assay was applied. Figure 4 shows the average values of % DNA migrating in the electric field to the “tail” of the comet, characterizing the level of destruction of the DNA molecule.

According to the comet assay, a relatively low level (5–6%) of nuclear DNA fragmentation is observed in male gametes of the sand dollars. However, after exposure of sperm with FMs leachates, the proportion of damaged DNA increases depending on the concentration of leachates from both types of experimental FMs (Figure 4A). In addition, it should be emphasized that at all dilutions used, leachates from weathered FMs had a greater effect compared with leachates from pristine FMs. At the same time, the average level of damaged DNA in *S. mirabilis* sperm after exposure to the maximum concentration of leachates from pristine and weathered FMs reached 15% and 20%, respectively. For a more detailed analysis, the comets that formed from sperm DNA before and after exposure with undiluted leachates from both FMs were grouped according to the degree of fragmentation at 3% intervals and are presented in Figure 4B. From the presented data, it can be seen that among the whole sample of native sperm of *S. mirabilis*, the upper limit of nuclear DNA damage does not exceed 24%. Whereas after exposure to leachates from pristine and weathered FMs, sperm with a higher degree of DNA damage (25–40%) were found among male gametes, with a total proportion of 18 and 28%, respectively.

#### 3.2.4. Success of Fertilization

**Figure 5:**
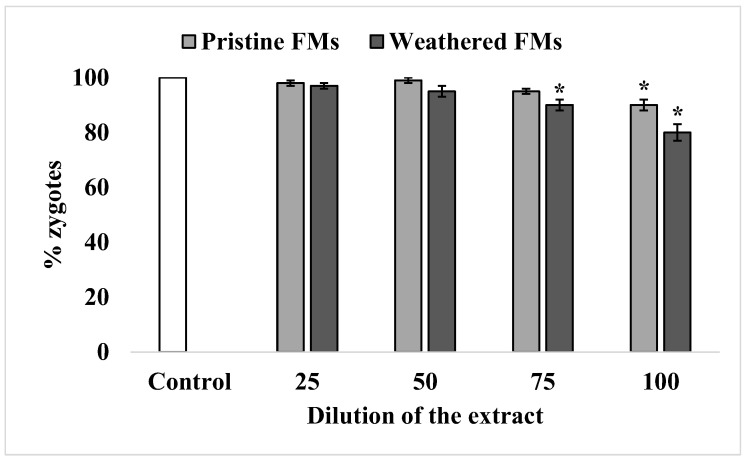
Fertilization rate of *S. mirabilis* eggs by sperm exposed to different concentrations of leachates from pristine and weathered face masks (mean ± standard deviation, n = 12); *—difference from control is significant at *p* < 0.05.

Under experimental conditions, the sperm of sand dollars *S. mirabilis* is characterized by high efficiency in oocyte fertilization, reaching almost 100%. After exposure to relatively low concentrations of leachates from both FMs types, sperm retained this ability unchanged. A significant decrease in sperm fertilization ability was observed only after exposure to relatively high concentrations of leachates (75% and higher). Nevertheless, the inhibitory effect was more pronounced with leachates from weathered FMs compared with leachates from pristine FMs (Figure 5).

## 4. Discussion

Against the background of the rapid spread of synthetic polymers, the introduction of huge masses of personal protective equipment, mainly FMs, into the environment in a relatively short period of time has further aggravated ecotoxicological problems [11,14,18,21,40]. At the same time, it has become evident that hydrobionts are most exposed to the risks of interacting with them and developing negative effects. Studies conducted at the height of the pandemic indicate the toxicological danger of leachates from FMs for primary producers, having an inhibitory effect on growth and photosynthetic processes in marine (*Phaeodactylum tricornutum*) and freshwater (*Scenedesmus obliquus*, *Chlorella* sp.) microalgae [25,26,41]. In addition, the components leached from FMs caused various harmful effects and significantly affected various stages of the reproductive process in key members of marine zooplankton, such as water flea *Daphnia magna*, fairy shrimp *Thamnocephalus platyurus* [42], brine shrimp *Artemia salina* [43], and copepod *Tigriopus japonicus* [6]. It was also found that in the bivalve *Mytilus galloprovincialis* that leachates from FMs induced oxidative stress and cyto- and genotoxicity [28].

This examples allow us to consider our experiments using male gametes of the sand dollars as a model of a real situation occurring in coastal waters under abundant pollution by face masks, which are a source of MPs and various chemicals [6,15,25,29,42,44,45]. The Far Eastern representative of the sand dollars *S. mirabilis* is one of the key species of benthic communities in the coastal waters of the Sea of Japan. For sand dollars, as for most littoral invertebrates, shallow coastal water areas, especially those with slow water exchange, are spawning grounds, as gametes are released directly into the sea water, where fertilization and larval development take place. In such places, there is a particularly high probability of direct interaction of germ cells and developing embryos with incoming terrigenous flows of chemical compounds leached from polymeric products, including FMs. Moreover, it is necessary to point out that, in contrast to somatic cells of adult individuals protected by tissue barriers and biochemical systems in gametes, especially in sperm, these systems are either absent or in an initial stage [46,47].

In our experiments, these biochemical features were clearly shown in the high sensitivity of *S. mirabilis* sperm to components easily leached from the plastic structure of FMs. It follows from the results obtained that leachates from pristine and weathered FMs reduced sperm viability; stimulated the accumulation of lipid peroxidation products, such as malondialdehyde (MDA); and significantly increased the DNA damage, showing a genotoxic effect.

From the biochemical point of view, the reduction in sand dollar sperm quality (resazurin test) after exposure to FMs leachates is the result of an inhibition in the activity of mitochondrial reductases, such as NADPH^+^ dehydrogenases, responsible for the transfer of electrons from NADPH^2^ to resazurin, reducing it to resorufin. Apparently, components leached from FMs caused disorganization of a wide range of biochemical systems and induced increased generation of reactive oxygen species (ROS) in experimental group sperm, which led to activation of lipid peroxidation (LPO) processes and oxidative DNA damage. This assumption based on biochemical markers has all grounds if we consider the high sensitivity of sperm to chemical agents exhibiting prooxidant and genotoxic properties. This is supported by experimental data showing the initiation of oxidative stress and DNA molecule damage when sand dollar sperm is exposed to metal oxide nanoparticles [48,49,50,51] and microplastic [52].

Apparently, biochemical disorders initiated by MPs components easily leached into the environment are more diverse and are not limited to the LPO activation and genotoxicity identified in our work. However, even when these markers were analyzed, serious biochemical abnormalities in experimental sperm were evident. There is every reason to believe that MDA accumulation because of oxidative destruction of lipids causes structural changes in the lipid matrix of membranes, which leads to a decrease in sperm motility [53,54]. It is necessary to pay special attention to the damages to DNA in the sperm detected in our experiments, as this biochemical marker has a prognostic character, especially from the point of view of the risks of developing long-term consequences that threaten the stability of populations, in particular, sand dollars.

Using the comet assay widespread in the genotoxicity studies of different pollutants [55], we have shown that after exposure of *S. mirabilis* sperm with leachates from pristine and weathered FMs, the average percentage of DNA in the comets tail increased depending on the concentration of leachates, indicating DNA damage in the sperm. The presence of a small level of DNA damage in control sperm can explain the accumulation of breaks naturally formed during gametogenesis [56,57]. However, in the analysis of sperm distribution according to the degree of DNA damage (with an interval of 3%), it can be seen that among the whole sample of experimental group sperm of the sand dollar *S. mirabilis*, a high degree of DNA damage (25–40%) was detected, the total proportion of which ranged from 18 to 28%, which showed a significant genotoxic effect. In this respect, the results confirm the previously presented separate experimental data indicating genotoxic effects of FMs leachates on the example of the marine bivalve *Mytilis galloprovincialis* [28] and a representative of the flora *Allium cepa* [58,59]. Moreover, leachates of polypropylene polymer, the main material of FMs, are also able to induce DNA damage [60].

However, despite the dose-dependent increase in DNA damage of sand dollar sperm, a significant decrease in egg fertilization success by these sperm was observed only at relatively high levels of DNA damage. Previously, we suggested the existence of a threshold value of DNA damage in sand dollar sperm, in which exceeding this leads to a decrease in fertilization success [7]. A lack of relationship between sperm DNA damage and fertilization success was also observed in other sea urchin species [61]. In marine mollusks such as mussels and oysters, short-term exposure of sperm cells to genotoxic agents also resulted in significant dose-dependent DNA damage, but sperm retained fertilizing ability [46,62]. A similar pattern was observed in fish sperm, which maintained fertilization success at high levels despite high levels of DNA damage induced by exposure to methyl methanesulfonate and diuron [63,64,65]. Based on our results and the literature data, it is logical to assume that sperm genome integrity is not a critical condition for successful oocyte fertilization. The comet assay allows us to assess genome destruction at early stages; therefore, the changes in the genetic apparatus of spermatozoa detected in our experiments may be of a delayed nature and are temporarily not shown in in integral functions. Taking into account that sperm DNA participates in the formation of a single genome with the oocyte, which ensures the development of the next generation, the integrity of gamete genomes is of paramount importance for the normal development of viable offspring. Therefore, the sperm DNA damage detected in our work may have distant consequences, increasing the probability of destructive processes in future generations of sand dollars.

In general, the results of our studies showed that the structure of face mask polymers contains labile components that can induce latent DNA defects. It should be emphasized that this is particularly strong in sperm exposed to leachates from weathered FMs. We did not define these components in extracts, the identification and characterization of which requires separate studies. Nevertheless, based on numerous literature data devoted to this issue, we can assert with high probability that the initiators of these effects are MPs, mainly PP, and a wide range of chemical compounds present in FMs [8,17,44,66,67,68,69,70]. As shown by modeling experiments, depending on the nature of the FM exposure, various sizes of PP microparticles ranging from 10^3^ to 10^6^ can be released into the aquatic environment [4,5,13,22,71]. Moreover, it has been suggested [69] that MPs found in FMs leachates are mainly generated already during the mask production process. Accordingly, on a biosphere scale, according to simple calculations [6], FMs produced in 2020 alone have become a source of more than 1370 trillion nano- and microparticles of plastic, generating them at a rate of up to 396 billion/day, which undoubtedly poses a serious threat to coastal inhabitants. In recent years, there has been a growing number of publications demonstrating the diverse toxic properties of MPs of various polymers, including PP, which are manifested at different levels of biological organization, including the molecular level [6,13,31,59,72,73,74].

In addition, chemicals such as incomplete polymerization products (mono-, di-, and oligomers) and chemical additives used during synthesis to impart specific physicochemical properties to polymers are present in various concentrations and easily leachable in synthetic polymers such as PP [8,45,66,69,70,75]. Because most of these chemical additives are present in high concentrations and are not covalently bonded to polymer chains, they can be released into the environment [76,77]. A clear example is the study by Biale and colleagues [78], who identified about 60 different chemical components that leach from aliphatic polymers, including PP. Arguably, each of these chemical compounds deserves increased attention and consideration of toxic properties. However, special attention should be paid to the presence of a variety of phthalic acid esters at elevated concentrations in FMs leachates, which are known to be carcinogens and exhibit reproductive toxicity by affecting sperm and embryo quality [79,80,81,82]. In addition to organic chemical compounds, mineral nanoparticles (Ag, Cu, TiO_2_) and high levels of heavy metals such as Pb, Cu, Zn, Cd, and Cr have been detected in FM fibers and leachates obtained by standard procedures [44,66,67,83]. From an ecotoxicological point of view, this group of inorganic components in FMs leachates is also of particular interest, as each of them exhibits genotoxic properties towards somatic cells and gametes of marine invertebrates [15,50,52,84]. It is logical to assume that in our experiments the above-mentioned organic and inorganic substances contributed to the negative impact on the sperm of the *S. mirabilis*.

Another result of our studies is that leachates from naturally weathered face masks exhibit negative biochemical shifts, including DNA damage, to a significantly greater extent than leachates from pristine FMs. We believe that in order to explain the reasons, we should refer to the known mechanisms of physicochemical degradation of polymers. In our experiments, face masks were subjected to a long-term (200 days) exposure to a complex of climatic factors, among which the leading role is solar activity (UV radiation). In fact, face masks composed of PP, which is confirmed by IR spectra, were subjected to long-term photodegradation in the open air. This polymer is known to be characterized by the highest rate of UV-initiated photooxidation among polyolefins [85].

In our case, during the time of stay in these conditions, the PP of FMs underwent significant degradation, which is confirmed by the appearance of new and changes in the existing bands in the IR spectra, especially in the carbonyl and hydroxyl bands. These changes in the FTIR spectra of weathered PP indicate the formation of O_2_-containing functional groups (ketoacids, aldehydes, and ketones) in the polymer structure as a result of H– and C–C bond breakage [86,87]. The level of oxidative degradation is quantitatively reflected in the increase in degradation indexes, mainly CI and HI (Table 1). It follows that the FM weathering process initiated the development of oxidative degradation and led to changes in the chemical composition of polymer chains of PP. In addition, such changes not only significantly reduce the hydrophobic properties of the polymer but also significantly affect the physical characteristics. It is shown that the oxidative degradation of PP during natural weathering or accelerated aging in laboratory conditions leads to a decrease in crystallinity with the formation of more flexible polymer chains and a loose polymer structure [88,89]. Modification of the physical state of polymer chains changes the forces holding endogenous chemicals that were added during synthesis or formed during oxidative degradation in the polymer structure. In fact, the polymer chains of PP LMs exposed to the environment undergo a set of physicochemical changes that lead to accelerated degradation and fragmentation, as well as promote a larger-scale release of nano- and microplastics and leaching of chemical components potentially harmful to the environment [4,22,78,90]. Therefore, it is logical to assume that the physicochemical processes occurring in the structure of the polymer of FM in the environment may be responsible for the increase in its toxic properties to the sand dollar sperm observed in our experiments. This explanation of our results is in good agreement with studies showing that leachates from weathered or artificially aged various polymers, including PP, are more toxic to living organisms compared with pristine plastic samples [28,59,91,92,93].

## 5. Conclusions

Nevertheless, our experimental results not only complement these insights but also suggest that FMs polymers contain components that affect gamete genome stability. Moreover, this negative effect is intensified in the process of physicochemical degradation that the FMs polymer structure undergoes in the environment. From the ecotoxicological point of view, taking into account that PP, which is the basis of FMs, decomposes within several decades, face masks, which appeared in the coastal zone, may pose a real threat for a long time to the reproduction of not only sand dollars, but also various littoral marine invertebrates.

## Figures and Tables

**Table 1:** Indexes of the content of functional groups in fragments of the outer layer of the FMs.

Index	Formula	Pristine FMs	Weathered FMs
CI	1850−1650/1500−1420 cm^−1^	0.19 ± 0.02	1.11 ± 0.15 *
HI	3400−3300/986−952 cm^−1^	0.08 ± 0.02	0.32 ± 0.11 *
COI	1200−1100/2940−2885 cm^−1^	0.23 ± 0.02	0.31 ± 0.04 *

*—difference from control is significant at *p* < 0.05.

## Data Availability

Data are contained within the article.

## Supplementary Materials

The following supporting information can be downloaded at: https://www.mdpi.com/article/10.3390/toxics13050372/s1, Figure S1: Graphic representation of the spermiotoxicity test procedure.

## References

[1] McHale M.E., Sheehan K.L. (2024). Bioaccumulation, transfer, and impacts of microplastics in aquatic food chains. J. Environ. Expo. Assess..

[2] Thompson R.C., Olsen Y., Mitchell R.P., Davis A., Rowland S.J., John A.W., McGonigle D., Russell A.E. (2004). Lost at sea: Where is all the plastic?. Science.

[3] Corcoran P.L., Biesinger M.C., Grifi M. (2009). Plastics and beaches: A degrading relationship. Mar. Pollut. Bull..

[4] Morgana S., Casentini B., Amalfitano S. (2021). Uncovering the release of micro/nanoplastics from disposable face masks at times of COVID-19. J. Hazard Mater..

[5] Mghili B., Analla M., Aksissou M. (2022). Face masks related to COVID-19 in the beaches of the Moroccan Mediterranean: An emerging source of plastic pollution. Mar. Pollut. Bull..

[6] Sun J., Yang S., Zhou G.J., Zhang K., Lu Y., Jin Q., Lam P.K.S., Leung K.M.Y., He Y. (2021). Release of microplastics from discarded surgical masks and their adverse impacts on the marine copepod *Tigriopus japonicus*. Environ. Sci. Technol. Lett..

[7] Chelomin V.P., Dovzhenko N.V., Slobodskova V.V., Mazur A.A., Kukla S.P., Zhukovskaya A.F. (2023). Expanded polystyrene-debris-induced genotoxic effect in littoral organisms. Toxics.

[8] Chen Q., Gao Z., Wu Y., Li H., Jiang J., Yang Y., Xu L., Shi H. (2023). Insight into chemical features of migrated additives from plastics and associated risks to estuarine ecosystem. J. Hazard Mater..

[9] De-la-Torre G.E., Dioses-Salinas D.C., Pizarro-Ortega C.I., Fernández-Severini M.D., Forero-López A.D., Dobaradaran S., Selvasembian R. (2023). Face mask structure, degradation, and interaction with marine biota: A review. J. Hazard Mater..

[10] Dioses-Salinas D.C., Pizarro-Ortega C.I., Fernández Severini M.D., Forero López A.D., Prieto G., Dobaradaran S., Kannan G., De-la-Torre G.E. (2023). Face mask exposure to environmental conditions: In situ physical and chemical degradation and interaction with marine organisms. Reg. Stud. Mar. Sci..

[11] Patrício Silva A.L., Prata J.C., Mouneyrac C., Barcelò D., Duarte A.C., Rocha-Santos T. (2021). Risks of Covid-19 face masks to wildlife: Present and future research needs. Sci. Total Environ..

[12] Liu Z., Wang J., Yang X., Huang Q., Zhu K., Sun Y., Van Hulle S., Jia H. (2022). Generation of environmental persistent free radicals (EPFRs) enhances ecotoxicological effects of the disposable face mask waste with the COVID-19 pandemic. Environ. Pollut..

[13] Cabrejos-Cardeña U., De-la-Torre G.E., Dobaradaran S., Rangabhashiyam S. (2023). An ecotoxicological perspective of microplastics released by face masks. J. Hazard. Mater..

[14] Morales I.D.G., Macusi E.D., Jondonero M.A.P., Guihawan J.Q., Bacosa H.P., Amparado R.F. (2023). Facemask: Protection or threat?. Mar. Pollut. Bull..

[15] Oliveira A.M., Patrício Silva A.L., Soares A.M.V.M., Barceló D., Duarte A.C., Rocha-Santos T. (2023). Current knowledge on the presence, biodegradation, and toxicity of discarded face masks in the environment. J. Environ. Chem. Eng..

[16] Prata J.C., Silva A.L.P., Walker T.R., Duarte A.C., Rocha-Santos T. (2020). COVID-19 pandemic repercussions on the use and management of plastics. Environ. Sci. Technol..

[17] De-la-Torre G.E., Dioses-Salinas D.C., Dobaradaran S., Spitz J., Nabipour I., Keshtkar M., Akhbarizadeh R., Tangestani M., Abedi D., Javanfekr F. (2022). Release of phthalate esters (PAEs) and microplastics (MPs) from face masks and gloves during the COVID-19 pandemic. Environ. Res..

[18] Shukla S., Khan R., Saxena A., Sekar S. (2022). Microplastics from face masks: A potential hazard post Covid-19 pandemic. Chemosphere.

[19] Hasan J., Shahriar S.I., Shahjahan M. (2023). Release of microfibers from surgical face masks: An undesirable contributor to aquatic pollution. Water Emerg. Contam. Nanoplastics.

[20] Yang Q., Yang S., Jiao Y. (2023). Assessing disposable masks consumption and littering in the post COVID-19 pandemic in China. Environ. Pollut..

[21] Wang Z., An C., Chen X., Lee K., Zhang B., Feng Q. (2021). Disposable masks release microplastics to the aqueous environment with exacerbation by natural weathering. J. Hazard. Mater..

[22] Saliu F., Veronelli M., Raguso C., Barana D., Galli P., Lasagni M. (2021). The release process of microfibers: From surgical face masks into the marine environment. Environ. Adv..

[23] Crisafi F., Smedile F., Yakimov M.M., Aulenta F., Fazi S., La Cono V., Martinelli A., Di Lisio V., Denaro R. (2022). Bacterial biofilms on medical masks disposed in the marine environment: A hotspot of biological and functional diversity. Sci. Total Environ..

[24] Pol W., Mierzynska K., Włodarczyka T., Hauschildb T., Zielinski P. (2023). No trophy for the trophy?—How lake trophy impacts bacterial assemblages of biofilm on microplastic. Ecohydrol. Hydrobiol..

[25] Sendra M., Rodriguez-Romero A., Yeste M.P., Blasco J., Tovar-Sánchez A. (2022). Products released from surgical face masks can provoke cytotoxicity in the marine diatom *Phaeodactylum tricornutum*. Sci. Total Environ..

[26] Das S., Chandrasekaran N., Mukherjee A. (2023). Unmasking effects of masks: Microplastics released from disposable surgical face masks induce toxic effects in microalgae *Scenedesmus obliquus* and *Chlorella* sp. Comp. Biochem. Physiol. Part C Toxicol. Pharmacol..

[27] Mohsen M., Zhang L., Sun L., Lin C., Wang Q., Liu S., Sun J., Yang H. (2021). Effect of chronic exposure to microplastic fibre ingestion in the sea cucumber *Apostichopus japonicus*. Ecotoxicol. Environ. Saf..

[28] Fonseca T., Edo C., Vilke J.M., Astudillo-Pascual M., Gonçalves J.M., Bebianno M.J. (2024). Impact of face masks weathering on the mussels *Mytilus galloprovincialis*. Water Emerg. Contam. Nanoplastics.

[29] Sendra M., Pereiro P., Yeste M.P., Novoa B., Figueras A. (2022). Surgical face masks as a source of emergent pollutants in aquatic systems: Analysis of their degradation product effects in *Danio rerio* through RNA-Seq. J. Hazard. Mater..

[30] Qualhato G., Cirqueira Dias F., Rocha T.L. (2024). Hazardous effects of plastic microfibres from facial masks to aquatic animal health: Insights from zebrafish model. Sci. Total Environ..

[31] Sun J., Zhu Y., Yin H., Yin J. (2024). The release of polypropylene plastic from disposable face masks in different water conditions and their potential toxicity in human cells. Environ. Pollut..

[32] Almeda R., Gunaalan K., Alonso-López O., Vilas A., Clérandeau C., Loisel T., Nielsen T.G., Cachot J., Beiras R. (2023). A protocol for lixiviation of micronized plastics for aquatic toxicity testing. Chemosphere.

[33] Rouillon C., Bussiere P.O., Desnoux E., Collin S., Vial C., Therias S., Gardette J.L. (2016). Is carbonyl index a quantitative probe to monitor polypropylene photodegradation?. Polym. Degrad. Stab..

[34] Campanale C., Savino I., Massarelli C., Uricchio V.F. (2023). Fourier transform infrared spectroscopy to assess the degree of alteration of artificially aged and environmentally weathered microplastics. Polymers.

[35] Kobayashi N. (1985). Marine pollution bioassay by sea urcin eggs, an attempt to enhance accuracy. Publ. Seto Mar. Biol. Lab..

[36] (2011). Biological Test Method: Fertilization Assay Using Echinoids (Sea Urchins and Sand Dollars).

[37] Dovzhenko N.V., Chelomin V.P., Mazur A.A., Kukla S.P., Slobodskova V.V., Istomina A.A., Zhukovskaya A.F. (2022). Oxidative Stress in Far Eastern Mussel Mytilus trossulus (Gould, 1850) Exposed to Combined Polystyrene Microspheres (PSs) and CuO Nanoparticles (CuO-NPs). J. Mar. Sci. Eng..

[38] Buege J.A., Aust S.D. (1978). Microsomal lipid peroxidation. Methods Enzymol..

[39] Knapp J.L.A., González-Pinzón R., Haggerty R. (2018). The resazurin-resorufin system: Insights from a decade of “smart” tracer development for hydrologic applications. Water Res..

[40] Mohanty M., Mohanty J., Dey S., Dutta K., Shah M.P., Das A.P. (2024). The face mask: A tale from protection to pollution and demanding sustainable solution. Emerg. Contam..

[41] Khoironi A., Hadiyanto H., Hartini E., Dianratri I., Joelyna F.A., Pratiwi W.Z. (2023). Impact of disposable mask microplastics pollution on the aquatic environment and microalgae growth. Environ. Sci. Pollut. Res. Int..

[42] Kalamaras G., Antonopoulou M., Beobide A.S., Triantafyllidis V., Dailianis S. (2024). Disposable face masks into aquatic media: Chemical and biological testing of the released compounds during the leaching process. Environ. Pollut..

[43] Pramanik D.D., Sharma A., Das D.K., Pramanik A., Kay P., Goycoolea F.M. (2024). Toxicological impacts of plastic microfibers from face masks on *Artemia salina*: An environmental assessment using Box-Behnken design. Mar. Environ. Res..

[44] López A.D.F., De-la-Torre G.E., Fernández Severini M.D., Prieto G., Brugnoni L.I., Colombo C.V., Dioses-Salinas D.C., Rimondino G.N., Spetter C.V. (2023). Chemical-analytical characterization and leaching of heavy metals associated with nanoparticles and microplastics from commercial face masks and the abundance of personal protective equipment (PPE) waste in three metropolitan cities of South America. Mar. Pollut. Bull..

[45] Bogush A.A., Kourtchev I. (2024). Disposable surgical/medical face masks and filtering face pieces: Source of microplastics and chemical additives in the environment. Environ. Pollut..

[46] Lewis C., Galloway T.S. (2008). Genotoxic damage in Polychaetes: A study of species and cell-type sensitivities. Mutat. Res./Genet. Toxicol. Environ. Mutagen..

[47] Lacaze E., Geffard O., Goyet D., Bony S., Devaux A. (2011). Linking genotoxic responses in *Gammarus fossarum* germ cells with reproduction impairment, using the Comet assay. Environ. Res..

[48] Gallo A., Manfra L., Boni R., Rotini A., Migliore L., Tosti E. (2018). Cytotoxicity and genotoxicity of CuO nanoparticles in sea urchin spermatozoa through oxidative stress. Environ. Int..

[49] Oliviero M., Schiavo S., Dumontet S., Manzo S. (2019). DNA damages and offspring quality in sea urchin *Paracentrotus lividus* sperms exposed to ZnO nanoparticles. Sci. Total Environ..

[50] Kukla S.P., Slobodskova V.V., Zhuravel E.V., Mazur A.A., Chelomin V.P. (2022). Exposure of adult sand dollars (*Scaphechinus mirabilis*) (Agassiz, 1864) to copper oxide nanoparticles induces gamete DNA damage. Environ. Sci. Pollut. Res. Int..

[51] Kukla S.P., Chelomin V.P., Mazur A.A., Slobodskova V.V. (2022). Zinc Oxide Nanoparticles Induce DNA Damage in Sand Dollar *Scaphechinus mirabilis* Sperm. Toxics.

[52] Mazur A.A., Chelomin V.P., Zhuravel E.V., Kukla S.P., Slobodskova V.V., Dovzhenko N.V. (2021). Genotoxicity of Polystyrene (PS) Microspheres in Short-Term Exposure to Gametes of the Sand Dollar *Scaphechinus mirabilis* (Agassiz, 1864) (Echinodermata, Echinoidea). J. Mar. Sci. Eng..

[53] Lu X.Y., Wu R.S. (2005). Ultraviolet damages sperm mitochondrial function and membrane integrity in the sea urchin *Anthocidaris crassispina*. Ecotoxicol. Environ. Saf..

[54] Lu X.Y., Wu R.S. (2005). UV induces reactive oxygen species, damages sperm, and impairs fertilisation in the sea urchin *Anthocidaris crassispina*. Mar. Biol..

[55] Jiang N., Naz S., Ma Y., Ullah Q., Khan M.Z., Wang J., Lu X., Luosang D.-Z., Tabassum S., Chatha A.M.M. (2023). An Overview of Comet Assay Application for Detecting DNA Damage in Aquatic Animals. Agriculture.

[56] Smith M.A., Fernandez-Triana J., Roughley R., Hebert D.N. (2009). DNA barcode accumulation curves for understudied taxa and areas. Mol. Ecol. Resour..

[57] Mahaye N., Thwala M., Cowan D.A., Musee N. (2017). Genotoxicity of metal based engineered nanoparticles in aquatic organisms: A review. Mutat. Res..

[58] Christudoss A.C., Kundu R., Dimkpa C.O., Mukherjee A. (2024). Time dependent release of microplastics from disposable face masks poses cyto-genotoxic risks in *Allium cepa*. Ecotoxicol. Environ. Saf..

[59] Christudoss A.C., Kundu R., Dimkpa C.O., Mukherjee A. (2025). Aging of disposable face masks in landfill leachate poses cyto-genotoxic risks to *Allium cepa*: Perils of uncontrolled disposal of medical waste. Plant Physiol. Biochem..

[60] Cappucci U., Proietti M., Casale A.M., Schiavo S., Chiavarini S., Accardo S., Manzo S., Piacentini L. (2024). Assessing genotoxic effects of plastic leachates in *Drosophila melanogaster*. Chemosphere.

[61] Manzo S., Schiavo S., Oliviero M., Toscano A., Ciaravolo M., Cirino P. (2017). Immune and reproductive system impairment in adult sea urchin exposed to nanosized ZnO via food. Sci. Total Environ..

[62] Akcha F., Spagnol C., Rouxel J. (2012). Genotoxicity of diuron and glyphosate in oyster spermatozoa and embryos. Aquat. Toxicol..

[63] Devaux A., Fiat L., Gillet C., Bony S. (2011). Reproduction impairment following paternal genotoxin exposure in brown trout (*Salmo trutta*) and Arctic charr (*Salvelinus alpinus*). Aquat. Toxicol..

[64] The Cardiolinc Network (2015). Long noncoding RNAs in cardiac development and ageing. Nat. Rev. Cardiol..

[65] Santos R., Palos-Ladeiro M., Besnard A., Porcher J.M., Bony S., Sanchez W., Devaux A. (2013). Relationship between DNA damage in sperm after ex vivo exposure and abnormal embryo development in the progeny of the three-spined stickleback. Reprod. Toxicol..

[66] Bussan D.D., Snaychuk L., Bartzas G., Douvris C. (2022). Quantification of trace elements in surgical and KN95 face masks widely used during the SARS-COVID-19 pandemic. Sci. Total Environ..

[67] Li A.S.H., Sathishkumar P., Selahuddeen M.L.W., Mahmood W.M.A., Abidin M.H.Z., Wahab R.A., Huri M.A.M., Abdullah F. (2022). Adverse environmental effects of disposable face masks due to the excess usage. Environ. Pollut..

[68] Sullivan G.L., Delgado-Gallardo J., Watson T.M., Sarp S. (2021). An investigation into the leaching of micro and nano particles and chemical pollutants from disposable face masks—Linked to the COVID-19 pandemic. Water Res..

[69] Wang X., Okoffo E.D., Banks A.P., Li Y., Thomas K.V., Rauert C., Aylward L.L., Mueller J.F. (2022). Phthalate esters in face masks and associated inhalation exposure risk. J. Hazard. Mater..

[70] Chang X., Wang W.X. (2023). Phthalate acid esters contribute to the cytotoxicity of mask leachate: Cell-based assay for toxicity assessment. J. Hazard. Mater..

[71] Jiang H., Su J., Zhang Y., Bian K., Wang Z., Wang H., Wang C. (2022). Insight into the microplastics release from disposable face mask: Simulated environment and removal strategy. Chemosphere.

[72] Chelomin V.P., Mazur A.A., Slobodskova V.V., Kukla S.P., Dovzhenko N.V. (2022). Genotoxic Properties of Polystyrene (PS) Microspheres in the Filter-Feeder Mollusk *Mytilus trossulus* (Gould, 1850). J. Mar. Sci. Eng..

[73] Tagorti G., Kaya B. (2022). Genotoxic effect of microplastics and COVID-19: The hidden threat. Chemosphere.

[74] Gong H., Li R., Li F., Guo X., Xu L., Gan L., Yan M., Wang J. (2023). Toxicity of nanoplastics to aquatic organisms: Genotoxicity, cytotoxicity, individual level and beyond individual level. J. Hazard. Mater..

[75] Leoni C., Majorani C., Cresti R., Marcello I., Berardi E., Fava L., Attias L., D’Ilio S. (2023). Determination and risk assessment of phthalates in face masks. An Italian study. J. Hazard. Mater..

[76] Gunaalan K., Fabbri E., Capolupo M. (2020). The hidden threat of plastic leachates: A critical review on their impacts on aquatic organisms. Water Res..

[77] Pires A., Cuccaro A., Sole M., Freitas R. (2022). Micro(nano)plastics and plastic additives effects in marine annelids: A literature review. Environ. Res..

[78] Biale G., La Nasa J., Mattonai M., Corti A., Castelvetro V., Modugno F. (2022). Seeping plastics: Potentially harmful molecular fragments leaching out from microplastics during accelerated ageing in seawater. Water Res..

[79] Radke E.G., Braun J.M., Meeker J.D., Cooper G.S. (2018). Phthalate exposure and male reproductive outcomes: A systematic review of the human epidemiological evidence. Environ. Int..

[80] Yost E.E., Euling S.Y., Weaver J.A., Beverly B.E., Keshava N., Mudipalli A., Arzuaga X., Blessinger T., Dishaw L., Hotchkiss A. (2019). Hazards of diisobutyl phthalate (DIBP) exposure: A systematic review of animal toxicology studies. Environ. Int..

[81] Xie H., Han W., Xie Q., Xu T., Zhu M., Chen J. (2022). Face mask-A potential source of phthalate exposure for human. J. Hazard. Mater..

[82] Shen L., Zhang C., Wang G., Fu X., Yang S., Wang J. (2025). High sperm DNA stainability might not be an accurate predictive indicator of male fertility and assisted reproductive technology outcomes. Front. Endocrinol..

[83] Verleysen E., Ledecq M., Siciliani L., Cheyns K., Vleminckx C., Blaude M.N., De Vos S., Brassinne F., Van Steen F., Nkenda R. (2022). Titanium dioxide particles frequently present in face masks intended for general use require regulatory control. Sci. Rep..

[84] Slobodskova V.V., Chelomin V.P., Kukla S.P., Mazur A.A. (2022). Copper Induced DNA Damage in the Gills of the Mussel *Mytilus trossulus* and Reversibility after Depuration. J. Mar. Sci. Eng..

[85] Gijsman P., Meijers G., Vitarelli G. (1999). Comparison of the UV-degradation chemistry of polypropylene, polyethylene, polyamide 6 and polybutylene terephthalate. Polym. Degrad. Stabil..

[86] Bandow N., Will V., Wachtendorf V., Simon F.G. (2017). Contaminant release from aged microplastic. Environ. Chem..

[87] Duan J., Li Y., Gao J., Cao R., Shang E., Zhang W. (2022). ROS-mediated photoaging pathways of nano- and micro-plastic particles under UV irradiation. Water Res..

[88] Guo X., Wang X., Zhou X., Kong X., Tao S., Xing B. (2012). Sorption of four hydrophobic organic compounds by three chemically distinct polymers: Role of chemical and physical composition. Environ. Sci. Technol..

[89] Wu X., Liu P., Huang H., Gao S. (2020). Adsorption of triclosan onto different aged polypropylene microplastics: Critical effect of cations. Sci. Total Environ..

[90] Du H., Huang S., Wang J. (2022). Environmental risks of polymer materials from disposable face masks linked to the COVID-19 pandemic. Sci. Total Environ..

[91] Liu P., Lu K., Li J., Wu X., Qian L., Wang M., Gao S. (2020). Effect of aging on adsorption behavior of polystyrene microplastics for pharmaceuticals: Adsorption mechanism and role of aging intermediates. J. Hazard. Mater..

[92] Pandi P., Madhuvandhi J., Priya K.K., Thiagarajan R., Gopalakrishnan S., Elumalai S., Thilagam H. (2022). Weathered polyethylene microplastics exposure leads to modulations in glutathione-S-transferase activity in fish. Front. Mar. Sci..

[93] Chelomin V.P., Istomina A.A., Mazur A.A., Slobodskova V.V., Zhukovskaya A.F., Dovzhenko N.V. (2024). New Insights into the Mechanisms of Toxicity of Aging Microplastics. Toxics.

