# Supplementary figures and images for: Leachate from Weathered Face Masks Increases DNA Damage to Sperm of Sand Dollars Scaphechinus mirabilis
Source: Toxics. 2025 May 4;13(5):372. doi: 10.3390/toxics13050372 (PMC12116056; doi:10.3390/toxics13050372)

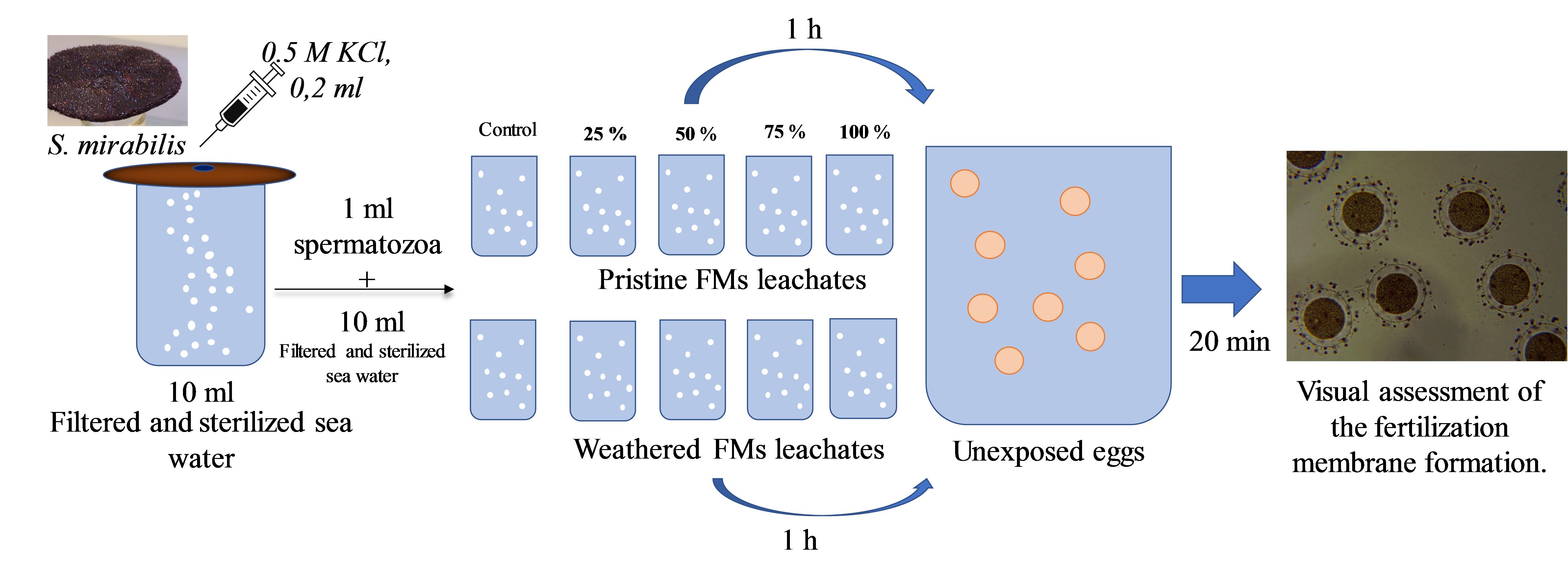

Supplement: Supplementary file 1 [file toxics-13-00372-s001.zip › toxics-3593218-supplementary.jpg]
